# Effect of sarpogrelate treatment on the prognosis after endovascular therapy for critical limb ischemia

**DOI:** 10.1007/s00380-013-0334-1

**Published:** 2013-03-14

**Authors:** Mitsuyoshi Takahara, Hideaki Kaneto, Naoto Katakami, Osamu Iida, Taka-aki Matsuoka, Iichiro Shimomura

**Affiliations:** 1 Department of Metabolic Medicine, Osaka University Graduate School of Medicine, 2-2 Yamadaoka, Suita, Osaka 565-0871 Japan; 2 Department of Internal Medicine, Kansai Rosai Hospital, Hyogo, Japan

**Keywords:** Sarpogrelate, 5-hydroxytryptamine type 2 antagonist, Critical limb ischemia, Amputation-free survival

## Abstract

5-Hydroxytryptamine type 2 antagonists are used to treat symptomatic peripheral arterial disease. However, it remains unknown as to whether the administration of sarpogrelate, a 5-hydroxytryptamine type 2 antagonist, improves the prognosis after endovascular therapy for critical limb ischemia (CLI). We performed a retrospective analysis using a database of 386 Japanese patients undergoing endovascular therapy for CLI. Sixty-seven patients were treated with sarpogrelate, and we compared their prognosis with that of an equal number of background-matched controls extracted from the population. The primary end point was the first event of either major amputation or death from any cause, and amputation-free survival was evaluated. The follow-up period was 21 ± 18 months (mean ± standard deviation), and 58 end points were observed. Patients treated with sarpogrelate had a significantly higher amputation-free survival rate than their matched controls (*P* = 0.036). The hazard ratio for the end point and its 95 % confidence interval was 0.57 (0.34–0.97). These results suggest that sarpogrelate treatment is associated with a favorable prognostic outcome in CLI patients undergoing endovascular therapy. Future prospective studies are required to investigate whether sarpogrelate treatment would improve the prognosis of CLI patients.

## Introduction

Critical limb ischemia (CLI) is the most advanced stage of peripheral arterial disease (PAD) characterized by chronic ischemic rest pain or tissue loss [1], and is associated with a poor prognosis for both life and limb and has a major impact on the quality of life [2]. Even after revascularization, a substantial number of patients are still likely to suffer a poor prognosis [3]. Therefore, it is of great interest as to whether there is any medication that can modify their prognosis after the revascularization procedures.

For the management of symptomatic PAD, clinical guidelines recommend naftidrofuryl, a 5-hydroxytryptamine type 2 (5-HT_2_) antagonist, as well as cilostazol, a phosphodiesterase III inhibitor [1]. Previous clinical trials revealed that both medications improve claudication in PAD patients [4, 5]. Recently, some studies have reported the promising effects of cilostazol on the prognosis of CLI patients [6], whereas the effects of 5-HT_2_ antagonists still remain to be investigated. Although naftidrofuryl is not clinically approved in Japan, another 5-HT_2_ antagonist, sarpogrelate, is available in clinical practice.

The aim of the current study was to investigate whether the administration of sarpogrelate improves the prognosis of Japanese patients undergoing endovascular therapy for CLI.

## Patients and methods

We used a database of 411 consecutive CLI patients undergoing endovascular therapy in Kansai Rosai Hospital, Hyogo, Japan, between April 2003 and April 2010, which did not include patients with thromboangiitis obliterans. The database comprised patients’ clinical characteristics at baseline and prospectively collected prognosis. Twenty-five patients (6 %) were excluded because of missing data. We included the remaining 386 patients (94 %), to retrospectively analyze the association between the administration of sarpogrelate and prognosis. The primary end point was the first event of either major amputation or death from any cause, and amputation-free survival was evaluated. Of the 386 patients, 67 (17 %) were treated with sarpogrelate. Since the clinical backgrounds were expected to be different between patients treated with and without sarpogrelate, we compared the prognosis of the 67 patients treated with sarpogrelate with that of an equal number of background-matched controls, extracted from the population, without sarpogrelate treatment. The investigation was approved by the ethics committee of Kansai Rosai Hospital, and informed consent was obtained from all patients.

Data are given as means and standard deviations for continuous variables or as percentages for dichotomous variables. Differences of continuous variables between two groups were evaluated by Student’s *t* test, whereas dichotomous variables between two groups were compared by the Fisher exact test, if not otherwise mentioned. Amputation-free survival rate was plotted using the Kaplan–Meier method, and the difference between groups was assessed by the log rank test. The Cox proportional hazards regression model was used to calculate the hazard ratio of sarpogrelate for the end point and its 95 % confidence interval. The model was also used to assess the interaction effect. A *P* value of less than 0.05 was considered statistically significant. All statistical analyses were performed using SPSS Statistics Version 19 (SPSS, Chicago, IL, USA), except for pair matching, which was performed using R version 2.13.0 (The R Foundation for Statistical Computing).

## Results

Of a total of 386 CLI patients, 67 (17 %) were treated with sarpogrelate at baseline and the remaining 319 (83 %) were not. Their clinical characteristics are shown in Table 1. The patients treated with sarpogrelate had a lower prevalence of diabetes mellitus (*P* = 0.034) and were less likely to be treated with cilostazol (*P* = 0.004). None of the patients were treated with prasugrel or had drug-eluting stents fitted for revascularization. When the matched controls were extracted and then compared with the 67 patients treated with sarpogrelate, no significant differences in clinical characteristics were observed between the two groups (Table 1). The follow-up period was 21 ± 18 months, and 58 end points were observed. The period wherein revascularization was performed was not significantly different between the patients treated with and without sarpogrelate, either before or after matching (*P* = 0.175 and 0.378, by Mann–Whitney *U* test).

**Table 1:** Clinical characteristics of CLI patients treated with or without sarpogrelate

	Patients with sarpogrelate (*n* = 67)	Patients without sarpogrelate	
		Overall (*n* = 319)	Matched controls (*n* = 67)
Male	40 (60 %)	217 (68 %)	45 (67 %)
Age (years)	71 ± 14	71 ± 10	71 ± 11
Body mass index (kg/m^2^)	21.0 ± 3.2	21.4 ± 3.2	21.0 ± 2.7
Nonambulatory state	21 (31 %)	134 (42 %)	24 (36 %)
Fontaine stage IV	57 (85 %)	258 (81 %)	59 (88 %)
Infection	15 (22 %)	68 (21 %)	16 (24 %)
Revascularization procedures			
Stent use	28 (42 %)	159 (50 %)	30 (45 %)
Self-expanding stent	27 (96 %)	136 (86 %)	29 (97 %)
Infrapopliteal revascularization	45 (67 %)	228 (71 %)	46 (69 %)
Comorbidities			
Coronary artery disease	21 (31 %)	115 (36 %)	29 (43 %)
Cerebrovascular disease	20 (30 %)	105 (33 %)	17 (25 %)
Diabetes mellitus	41 (61 %)	239 (75 %)*	47 (70 %)
Hypertension	60 (90 %)	272 (85 %)	60 (90 %)
Dyslipidemia	49 (73 %)	246 (77 %)	53 (79 %)
Smoking	39 (58 %)	188 (59 %)	42 (63 %)
Hemodialysis	29 (43 %)	147 (46 %)	27 (40 %)
Antiplatelet therapy			
Aspirin	55 (82 %)	282 (88 %)	56 (84 %)
Thienopyridines	17 (25 %)	113 (35 %)	18 (27 %)
Cilostazol	29 (43 %)	200 (63 %)*	33 (49 %)
More than one agent	36 (54 %)	220 (69 %)*	40 (60 %)

Data are mean ± standard deviation or *n* (%)

* *P* < 0.05 compared with patients treated with sarpogrelate

**Fig. 1:**
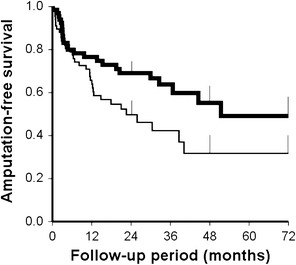
Amputation-free survival in CLI patients treated with and without sarpogrelate. Amputation-free survival rates were estimated by the Kaplan–Meier method. Patients treated with sarpogrelate (*thick line*) had a higher amputation-free survival rate than the matched controls (*thin line*) (*P* = 0.036 by log rank test). *Error bars* represent standard errors

Figure 1 shows the amputation-free survival rate of patients treated with sarpogrelate and their matched controls. The patients treated with sarpogrelate had a significantly higher rate of amputation-free survival than the matched controls (*P* = 0.036 by log rank test). The hazard ratio for the end point and its 95 % confidence interval was 0.57 (0.34–0.97). No interaction effect was observed between sarpogrelate treatment and diabetes mellitus (*P* = 0.836).

## Discussion

The current retrospective study suggests that the administration of sarpogrelate is associated with a higher amputation-free survival rate in Japanese patients undergoing endovascular therapy for CLI.

Management of cardiovascular ischemic diseases is a challenging issue in clinical practice [7–15]. Management of CLI is no exception; patients with CLI still have a poor prognosis even after revascularization. It is of clinical interest as to whether there is any treatment that can improve their prognosis [16–18]. Sarpogrelate is a 5-HT_2_ antagonist and is now clinically approved for symptomatic PAD in Japan. Although its mechanisms of action are not yet fully understood, previous reports showed that sarpogrelate reduces platelet aggregation [19, 20], improves endothelial function [21, 22], and enhances peripheral circulation [23, 24]. In addition, some clinical trials have demonstrated its favorable effects on cardiovascular diseases [25] and skin ulcers [26], as well as intermittent claudication [27]. On the other hand, the potential effects of sarpogrelate on the prognosis of CLI remain unrevealed. In the current study we performed a retrospective analysis to investigate the association of sarpogrelate treatment with the prognosis of CLI after endovascular procedures.

In the current study population, sarpogrelate was administered to 17 % of the CLI patients undergoing endovascular therapy. The patients with sarpogrelate treatment were less likely to be treated with cilostazol (*P* = 0.004), which would be clinically explainable. Both sarpogrelate and cilostazol are expected to relieve PAD-related symptoms, and clinicians would reasonably assume the two medications as equals in the management of symptomatic PAD [27]. Therefore, it would be no surprise if patients already treated with one of the medications are not treated with the other.

Another difference in clinical characteristics between patients treated with and without sarpogrelate was the prevalence of diabetes mellitus. Patients treated with sarpogrelate had a lower prevalence of diabetes mellitus (*P* = 0.034); in other words, diabetic patients were less likely to be treated with sarpogrelate. Diabetic patients often suffer decreased pain sensation [28, 29], so CLI patients with diabetes mellitus might not feel foot pain as severely as those without diabetes. Sarpogrelate is expected to relieve the symptoms related to PAD. It would be no surprise that the administration of sarpogrelate was associated with the severity of symptoms. In the diabetic CLI patients, less frequent complaints of pain might lead to less frequent administration of sarpogrelate, although no data were available about the severity of neuropathy and symptoms in the current study population.

To minimize the prognostic influence of these different clinical backgrounds, we used matched controls to perform the comparison. As a result, the treatment with sarpogrelate was significantly associated with a favorable prognostic outcome (*P* = 0.036). However, the current analysis was retrospectively performed, which is its major limitation. The decision on the administration of sarpogrelate was left to the judgment of each doctor. We could not obtain data about the reason why each patient was treated with sarpogrelate. Covariates that were not taken into consideration in the current study might affect the prognostic findings. Whether the administration of sarpogrelate can improve prognostic outcome, therefore, still remains unrevealed. Nonetheless, the current findings raise our clinical hope for prognostic improvement by way of sarpogrelate treatment in CLI patients after endovascular therapy. Future prospective controlled trials are required to confirm the efficacy of sarpogrelate treatment for a favorable prognostic outcome.

In conclusion, sarpogrelate treatment was associated with a favorable prognostic outcome in Japanese CLI patients undergoing endovascular therapy. Future prospective studies are required to investigate whether sarpogrelate treatment would improve the prognosis of CLI patients.
